# Awareness and use of the Canadian computed tomography head rule for mild head injury patients among Chinese emergency physicians

**DOI:** 10.12669/pjms.294.3469

**Published:** 2013

**Authors:** Xin Huang, Jian-cang Zhou, Kong-han Pan, Hong-chen Zhao

**Affiliations:** 1 Xin Huang, Department of Neurosurgery, 1st Affiliated Hospital, Zhejiang University School of Medicine, Hangzhou, Zhejiang, China.; 2 Jian-cang Zhou, Department of Critical Care Medicine, Sir Run Run Shaw Hospital, Zhejiang University School of Medicine, Hangzhou, Zhejiang, China.; 3 Kong-han Pan, Department of Critical Care Medicine, Sir Run Run Shaw Hospital, Zhejiang University School of Medicine, Hangzhou, Zhejiang, China.; 4 Hong-chen Zhao, Department of Critical Care Medicine, Sir Run Run Shaw Hospital, Zhejiang University School of Medicine, Hangzhou, Zhejiang, China.

**Keywords:** Guideline, Mild head injury, Computed tomography, Emergency department

## Abstract

***Objective:*** Computed tomography (CT) scan has been an increasingly essential diagnostic tool for emergency physicians (EPs) to triage emergency patients. Canadian computed tomography Head Rule (CCHR) had been established and widely used to spare patients with mild head injury from unnecessary radiation. However, the awareness of CCHR and its actual utilization among Chinese EPs were unknown. This survey was to investigate the awareness and use of CCHR and their associated characteristics among Chinese EPs.

***Methods:*** Questionnaire was randomly sent to EPs from different Chinese hospitals. Surveyed EPs were asked how well they know about the CCHR and how often they use the CCHR to guide head CT use. Association between the awareness and utilization of CCHR and the physicians’ characteristics were analyzed using repeated-measures logistic regression.

***Results:*** About 41.7% of the total 247 responders noted they “very familiar” or “somewhat familiar” with CCHR while the utilization rate was 24.7%. With respect to the most important underlying barriers for the use of CCHR, approximate half (48.5%) cited “fear of malpractice” as the leading cause. “Received specific training regarding radiation dose of CT” was the significant predicting factor both for the awareness (OR 5.87; 95% CI, 3.08-11.21) and the use (OR 6.10, 95% CI, 2.91-12.80) of CCHR.

***Conclusions:*** Fear of malpractice and lack of radiation risk knowledge were two main barriers to apply CCHR in the request of CT for patients with mild head injury. Furthermore, EPs with specific training about radiation risk of CT were more likely to know and use of CCHR.

## INTRODUCTION

Computed tomography (CT) scan is an integral diagnostic tool in the emergency department due to its widely accessibility and diagnostic accuracy.^1^ To some extend, CT is currently an indispensable tool to triage and manage patients with traumatic head injury because the consequences of missing a clinically important problem are potentially life-threatening. Hence, number of CT scans requested by emergency physicians (EPs) is increasing at an astonishing rate.^2^^,^^3^ However, yields were extremely low if patients with minor head injury were mandatory to have CT scans without any selection.^4^ Given that CT is expensive and has been associated with increased cancer risk due to radiation exposure, there are increasing concerns that the use of head CT for emergency patients with minor head injury should be justified. 

Under this circumstance, the Canadian computed tomography Head Rule (CCHR) was derived in 2001, comprising five high-risk criteria (including GCS (Glasgow Coma Scale) score <15 at 2 hours after injury, suspected open or depressed skull fracture, any sign of basal skull fracture, vomiting ≥2 episodes, and age ≥ 65 years) and two medium-risk criteria (including amnesia before impact ≥ 30 minutes and dangerous mechanisms such as pedestrian struck by motor vehicle or fall from elevation ≥3 feet or 5 stairs) to determine the need for CT in patients with minor head injury.^4^ Patients without these high-risk criteria can be safely managed but spared risk of unnecessary radiation. In 2005, it underwent prospective external validation among 1,822 patients and demonstrated 100% sensitivity and a specificity of 50.6% and 76.3% for predicting clinically important brain injury and need for neurosurgical intervention respectively.^5^ Utilization of CCHR would decrease CT scan by 37%. Therefore, the CCHR, combined with EPs’ judgment, seems to be the best available option to guide CT scan use in patients with minor head injury. 

Nevertheless, in our previous study,^6^ head CT accounted for approximate 70% to 80% of the total CT utilization between 2005 and 2008 because vast majority of the emergency visits with mild head injury underwent head CT. Among them, some could be spared should the EPs used the CCHR to determine whether patients with mild head injury really need a CT. Thus, it is very interesting to study how well the Chinese EPs know about the CCHR and how often they use the CCHR to guide head CT use in managing alert, stable patients with minor head injury, yet few empirical data are available regarding this. Therefore, the primary goal of this survey was to provide a representative picture of the current awareness and use of CCHR among Chinese EPs, as well as to explore the associated underlying factors. 

## METHODS

We conducted a self-administrated e-mail and postal survey of EPs from different Chinese hospitals. The institutional review board of first Affiliated Hospital, Zhejiang University School of Medicine approved the study protocol and waived from the need for a consent form.

We surveyed EPs who were randomly selected from the participant directory of the Annual Emergency Medicine Conference. A fifteen-question questionnaire (Appendix) was sent to them by either e-mail or postal mail with a prepaid addressed envelope. No incentives were provided to facilitate the response. Participants were encouraged to distribute this questionnaire to their colleagues or friends also working as EPs. This method of distribution did not allow us to report the response rate. Non-respondents were sent a minimum of one reminder four weeks later. The survey assessed overall characteristics of the participants and their emergency departments, general knowledge about radiation and their use of CCHR.

Descriptive data were reported as number and percentage. To facilitate the statistical analysis, we regarded respondents who reported that they know the CCHR “very familiar” and “somewhat familiar” as familiar with CCHR (“1”) and those who reported that they “not very familiar” and “not at all familiar” as not familiar with CCHR (“0”). Similarly, we coded those use the rule ‘‘always’’ or ‘‘most of the time’’ as users (“1”) and those use the rule ‘‘sometimes’’ or “never’’ as nonusers (“0”).

To identify potential correlation between hospitals’ and EPs’ characteristics and awareness and use of the CCHR, binary logistic regression analysis was performed using whether familiar with CCHR (” 1” or “0”), and whether use CCHR (” 1” or “0”) to guide the CT use for alert, stable patients with mild head injury respectively as the dependent variable and physicians’ demographics, emergency departments’ characteristics, availability of CT and radiation risk education as the independent variables. The generalized estimating equation regression model was used to account for the effect of clustering of physicians among the same hospital, which may make their responses not independently. Odds ratios and their 95% confidence intervals (95% CIs) were calculated. The Hosmer-Lemeshow goodness-of-fit test was used to test the multivariate logistic regression model fit. Statistical analysis was performed, using SPSS 16.0 (Chicago, Ill, USA). Significance was defined as a *P *value <0.05. 

## RESULTS

Two hundreds and forty-seven physicians returned their questionnaires. Physician demographic, professional and hospital setting characteristics are summarized in Table-I. The vast majority of them were male (70.9%), had an around the clock accessible head CT scan (93.9%), and worked more than 5 years as EPs. More than half of the responders worked at a teaching hospital. Only around one sixth of them had a primary training of emergency medicine, while physicians who were initially trained in medicine or surgery were similar around 40%. In terms of the average annual emergency visits volume, nearly half had an annual visit volume around 50,000-100,000, followed by 32.8% participants came from emergency departments with a yearly volume >100,000. Meanwhile, more than half surveyed physicians with an average >1000 monthly trauma visits. Surprisingly, only one third had received some specific training about the radiation risk of medical imaging. Although 83% responders believed lifetime risk of cancer could be increased by CT scan, less than half (44.9%) knew the exact corresponding radiation dose of a CT scan equals to chest radiography. With respect to the CCHR, only 41.7% of the responders noted they “very familiar” or “somewhat familiar”, while the utilization rate was as low as 24.7%. Interestingly, when those physicians who did not currently use the CCHR were asked to whether they would consider use it in the future, 68.3% of them gave a positive answer. When it comes to the most important underlying barriers for the use of CCHR, approximate half (48.5%) cited “fear of malpractice” as the leading cause, followed by “pressure from administration to order more examinations” and “lack of knowledge about the radiation risk of CT”, around 29.5% and 27.3% respectively. 

**Table-I:** Demographic and professional characteristics of the surveyed emergency physicians

*Characteristics*	*Number (%)*
*Years of practice*	
>15	9 (3.6%)
11-15	62 (25.1%)
6-10	115 (46.6%)
≤5	61 (24.7%)
*Current professional rank*	
Attending	65 (26.3%)
Fellow	122 (49.4%)
Resident	60 (24.3%)
*Primary training *	
Medicine	109 (44.1%)
Surgery	98 (39.7%)
Emergency medicine	40 (16.2%)
Male gender	175 (70.9%)
Teaching hospital	139 (56.3%)
*Approximate annual ED visit volume*	
<50,000	50 (20.2%)
50,000-100,000	116 (47.0%)
>100,000	81 (32.8%)
*Approximate monthly trauma visit volume*	
<500	57 (23.1%)
500-1000	52 (21.1%)
>1000	138 (55.9%)
Head CT available anytime	232 (93.9%)
Received specific training regarding radiation dose of CT	88 (35.6%)
Familiar with CCHR	103 (41.7%)
Routine apply CCHR when request of CT for patients with mild head injury	61 (24.7%)

ED, emergency department, CT, computed tomography, CCHR, Canadian Computed Tomography Head Rule

**Table-II:** Association between emergency physicians’ characteristics and familiar with CCHR

*Characteristics*	*OR*	*95% CI*	*P*
*Years of practice (vs. ≤5 years)*			
>15	1.27	0.09-18.95	0.861
11-15	0.89	0.10-7.95	0.914
6-10	0.84	0.11-6.31	0.869
*Current professional rank (vs. resident)*			
Attending	0.96	0.14-6.70	0.967
Fellow	0.99	0.11-8.84	0.992
*Primary training (vs. emergency medicine)*			
Medicine	1.15	0.48-2.79	0.752
Surgery	1.37	0.54-3.52	0.507
Male gender	1.41	0.72-2.74	0.314
Teaching hospital	0.85	0.42-1.70	0.639
*Approximate annual ED visit volume (vs. * *<50,000* *)*			
50,000-100,000	1.50	0.41-5.47	0.542
>100,000	2.25	0.50-10.04	0.288
*Approximate monthly trauma visit volume (vs. * *<500* *)*			
500-1000	1.44	0.41-5.11	0.569
>1000	1.16	0.33-4.15	0.819
Head CT available anytime	0.70	0.16-3.10	0.642
Received specific training regarding radiation dose of CT	5.87	3.08-11.21	<0.001

CCHR, Canadian Computed Tomography Head Rule, ED, emergency department, CT, computed tomography, OR, Odds Ratio, CI, confidence interval.

**Table-III:** Association between emergency physicians’ characteristics and use of CCHR

*Characteristics*	*OR*	*95% CI*	*P*
*Years of practice (vs. ≤5 years)*			
>15	0.18	0.01-5.47	0.326
11-15	0.27	0.02-3.43	0.314
6-10	0.23	0.02-2.32	0.213
*Current professional rank (vs. resident)*			
Attending	3.66	0.29-46.32	0.317
Fellow	4.13	0.45-38.13	0.212
*Primary training (vs. emergency medicine)*			
Medicine	1.29	0.44-3.81	0.643
Surgery	1.86	0.59-5.86	0.289
Male gender	2.75	1.18-6.41	0.020
Teaching hospital	0.82	0.36-1.85	0.628
*Approximate annual ED visit volume (vs. * *<50,000* *)*			
50,000-100,000	0.72	0.12-4.21	0.716
>100,000	0.87	0.12-6.26	0.888
*Approximate monthly trauma visit volume (vs. * *<500* *)*			
500-1000	6.52	1.06-40.06	0.043
>1000	5.32	0.80-35.51	0.085
Head CT available anytime	0.88	0.07-10.47	0.918
Received specific training regarding radiation dose of CT	6.10	2.91-12.80	<0.001

CCHR, Canadian Computed Tomography Head Rule, ED, emergency department, CT, computed tomography, OR, Odds Ratio, CI, confidence interval.

In order to elucidate the influence of EPs, professional characteristics on their awareness and use of CCHR during practice, we coded practice years, current professional rank, primary training, average annual emergency department visits volume and monthly trauma visits volume as dummy variables. Therefore, practice years of “≤5 years”, current “resident physician”, primary training in “emergency medicine”, annual volume “<50,000”, and monthly trauma volume “<500” were coded as reference, or “0”. Adjusted odds ratios of awareness and use of CCHR were derived using a multivariate model and were shown in Table II and III respectively. Hosmer-Lemeshow goodness-of-fit test showed *P*=0.390 and *P*=0.959 respectively, which indicate models fit. “Received specific training regarding radiation dose of CT” (adjusted odds ratio [OR] 5.87; 95% CI, 3.08-11.21) was the only significant predicting factor for the awareness of CCHR among the responded physicians. As for the utilization of CCHR, monthly trauma visit volume of “500-1000” (OR 6.52, 95% CI, 1.06-40.06), male gender (OR 2.75, 95% CI, 1.18-6.41) and “Received specific training regarding radiation dose of CT” (OR 6.10, 95% CI, 2.91-12.80) were significant predictor for the use of CCHR during practice.

## DISCUSSION

The current survey demonstrated the awareness and utilization rate of CCHR among Chinese EPs was 41.7% and 24.7%. Approximate half (48.5%) cited “fear of malpractice” as the root cause for not apply CCHR during practice. “Received specific training regarding radiation dose of CT” was the significant predicting factor for both the awareness of CCHR (OR 5.87; 95% CI, 3.08-11.21) and the use of CCHR during practice (OR 6.10, 95% CI, 2.91-12.80).

CT is widely accepted as an effective diagnostic modality to detect rare but clinically significant intracranial injuries in patients suffering minor head injury. As such, it has been increasingly utilized as a routine test for patients with mild head injury.^1^ In a large Chinese tertiary hospital, we found CT utilization increased from 9.8% in 2005 to 13.9% in 2008 for emergency department visits.^6^ Moreover, variation in CT use for patients with head injury between hospitals and interphysicians were significantly.^7^ Consequently, the number of CT scans per trauma patient has more than doubled over 6 years.^8^ Not surprisingly, the radiation exposure has increased in trauma patients over time. On the other hand, imaging has been the highest rate of growth among all healthcare services cost between 2000 and 2006, increasing at 17% per year.^9^ Thus, unnecessary exposure to ionizing radiation by overuse of head CT has raised concerns for patients, health care providers and regulators. In order to allow the EPs to standardize and be more selective in their use of CT but without compromising care of patients with minor head injury, CCHR was derived in 2001,^4^ and prospective validated externally not only in North American but also in other countries,^5^^,^^10^^,^^11^ suggesting that reducing the usage rate of CT for emergency patients with minor head injury to as low as 62.4% was possible and safe.^12^

A similar survey of awareness and use of the CCHR conducted in Canada, Australasia, the United Kingdom and the United States previously demonstrated that awareness of CCHR ranged from 31% in United States to 86% in Canada, while the utilization rate varied from 12% in United States to 57% in Canada.^13^ In our survey, as expected, the rate of awareness and use of CCHR among Chinese EPs were much lower, at 41.7% and 24.7% respectively. Consistent with other guidelines, it takes years to translate into actual practice. As we know, barriers of successful implementation of the evidenced-based guideline include the guideline itself, institutional factors, characteristics of both the provider and the patient that influenced. We evaluated potential barriers in our survey, almost half respondents stated “fear of malpractice” as the leading cause, followed by “pressure from administration to order more examinations” and “lack of knowledge about the radiation risk of CT”, around 29.5% and 27.3% respectively. This is consistent with the main causes of imaging overuse.^14^^,^^15^ Since the physicians-patients relationship getting increasingly worse, defensively ordering CT scans for minor diseases is common.^16^ To order head CT for any patients with minor head injury so as to avoid malpractice as much as possible seems intuitively appealing. In some setting, defensive medicine accounts for approximate 1 in 5 examinations.^14^ On the other hand, ignorance of radiation risk is widespread. In this study, more than half respondents underestimated the corresponding radiation dose of a CT scan compared to chest radiography. Thus, a vicious cycle to order much CT is precipitated by knowledge gap of the ordering provider, a widespread economic incentive of the hospital and patients demand on the assumption that more information is better. Interestingly, “Received specific training regarding radiation dose of CT” was the significant predicting factor both for the awareness and use of CCHR in our study. Thus, by understanding the attitudes of EPs toward the CCHR and the underlying barriers for use, we may be in a more strategic position to move forward with quality improvement activities, such as adoption of a comprehensive approach that targets physicians’ education and improves legislation support, which will help to reduce the over-reliance on CT imaging for head injury patients. 

Our study has several limitations. First, our study only assessed the awareness and use of CCHR among Chinese emergency physicians, which may raise the concerns that there may be other guideline rather than CCHR being used in some institution such as National Institute for Clinical Excellence and New Orleans Criteria for CT Scanning.^10^^,^^13^ However, we asked the physicians to specify any guidelines they are currently using to guide the use of head CT for patients with mild head injury, yet none of them mentioned any other guidelines. Second, given the self-administered nature of the survey, we cannot be certain what the respondents reported is a true reflection of their actual daily practice. Third, our method of recruitment that encouraging physicians to distribute between colleagues meant that we were unable to obtain a denominator for calculating a response rate.

In summary, our survey showed fear of malpractice and lack of radiation risk knowledge were two main barriers to apply CCHR in the request of CT for patients with mild head injury. Further, EPs with specific training about radiation risk of CT were more likely to know and use of CCHR. A better understanding of the factors related to awareness and use of EPs’ decision rules will enhance our understanding of knowledge translation and facilitate strategies to enhance dissemination and implementation of CCHR among Chinese EPs.
